# A Comparative Study of Pancreatic Acinar Cell Carcinoma: A Case–Control Study

**DOI:** 10.1002/hsr2.70633

**Published:** 2025-04-10

**Authors:** Bor‐Shiuan Shyr, Ting‐Chung Chen, Shin‐E. Wang, Shih‐Chin Chen, Yi‐Ming Shyr, Bor‐Uei Shyr

**Affiliations:** ^1^ Division of General Surgery, Department of Surgery and Therapeutic and Research Center of Pancreatic Cancer Taipei Veterans General Hospital Taipei Taiwan (ROC); ^2^ National Yang Ming Chiao Tung University Taipei Taiwan (ROC)

**Keywords:** amylase, lipase, pancreatic acinar cell carcinoma, pancreatic head adenocarcinoma

## Abstract

**Background and Aims:**

Pancreatic acinar cell carcinoma (PACC) is rare. This study aims to elucidate its clinical features and survival outcomes.

**Methods:**

Patients diagnosed with PACC were enrolled. A comparison between PACC and pancreatic ductal adenocarcinoma (PDAC) patients was conducted following propensity score matching (PSM).

**Results:**

There were 11 resectable and nine unresectable PACC. The majority (60%) of PACC cases were located in the pancreatic head, with a median tumor size of 5.9 cm. Elevated serum lipase level was observed in 64.3% of cases. Regional lymph node involvement was found in 65.0%. The median survival was 20.0 months for resectable PACC patients compared to 6.7 months for unresectable cases. The 1‐, 3‐, and 5‐year survival rates were 100%, 49.1%, and 32.0%, respectively, for resectable PACC patients, while for unresectable cases, they were 33.3%, 0%, and 0%. Resectable PACC patients exhibited lower rates of lymph node involvement (36.4% vs. 66.1%), lymphovascular invasion (LVI), 36.4% versus 72.8%, and perineural invasion (PNI), 45.5% versus 86.0%, compared to PDAC. Following PSM, there was no significant difference in survival between resectable PACC and PDAC.

**Conclusion:**

PACC is associated with lower rates of lymph node involvement, LVI, and PNI, which might attribute to superior outcome when compared with PDAC reported in the literature. However, there is no survival difference between resecrable PACC and PDAC after PSM.

## 1 Introduction

Pancreatic acinar cell carcinoma (PACC) is a rare malignant tumor characterized by distinctive clinical, molecular, and morphological features. It consists of cells bearing morphological resemblance to acinar cells and demonstrating evidence of exocrine enzyme synthesis by the neoplastic cells. Despite acinar cells comprising 82% of the pancreas, PACC accounts for only 0.2%–4.3% of all pancreatic carcinomas [1, 2, 3]. A notable clinical characteristic is the hypersecretion of lipase without an associated elevation in serum amylase level. This lipase hypersecretion can lead to pancreatic panniculitis, characterized by ill‐defined erythematous subcutaneous nodules primarily on the legs. Additionally, it may manifest with arthropathy, synovitis, osteolytic bone lesions, and polyserositis. The occurrence of “Schmid's triad” [4], a syndrome featuring subcutaneous fat necrosis, polyarthralgia, and eosinophilia due to increased serum lipase, is typical of PACCs but is generally rare [5].

Understanding the morphologic and immunohistochemical characteristics of this lesion is crucial for precise clinical diagnosis. PACC typically presents with nonspecific signs or symptoms such as abdominal pain, weight loss, and abdominal mass; jaundice is less common than in pancreatic ductal adenocarcinoma (PDAC). Compared to PDAC, PACC is associated with a more favorable prognosis. However, despite recommendations for radical surgery in PACC patients without distant metastases, the recurrence rate remains high [1, 2, 3, 6, 7, 8, 9, 10, 11].

Currently, our understanding of PACC is limited by the absence of large‐scale clinical studies. Most of our knowledge about PACC is derived from case reports, a few case series, or meta‐analysis studies. The rarity of PACC poses challenges, leaving many questions unanswered regarding its clinical features and survival outcomes. This study aims to investigate the clinical characteristics, metastatic sites, and treatment approaches by comparing resectable and unresectable cases of PACC. Additionally, to elucidate the prognosis of PACC, a comparison between resectable PACC and PDAC patients after propensity score matching (PSM) is also conducted.

## 2 Materials and Methods

### 2.1 Patients and Clinical Characteristics

Data from patients with pathologically confirmed PACC were retrieved from pathology databases spanning 1999–2024. Additionally, as the control group for comparison of prognosis, patients with resectable PDAC undergoing pancreaticoduodenectomy and distal pancreatectomy were identified from a prospectively‐collected computer database. Approval for this study was obtained from the Institutional Review Board (IRB) of Taipei Veterans General Hospital (IRB‐TPEVGH No. 2024‐05‐011AC), and it adhered to the ethical guidelines for human studies proposed by our IRB. Due to the anonymized and retrospective nature of this cohort study, the requirement for informed consent was waived. Clinical characteristics, including sex, age, location of PACC, tumor size, clinical presentations, and serum markers such as lipase, amylase, carbohydrate antigen 19‐9 (CA 19‐9), carcinoembryonic antigen (CEA), and α‐fetoprotein (AFP), were evaluated and compared between resectable and unresectable PACCs. Metastatic sites, treatment modalities, and survival outcomes were investigated for both resectable and unresectable PACC groups. The radicality of resection was categorized into three groups based on the resection margin status: R0, indicating a resection without any visible or microscopic evidence of cancer at the resection margin, with a margin > 1 mm; R1, indicating a resection with no visible evidence but microscopic presence of cancer at the resection margin, with a margin ≤ 1 mm; and R2, indicating a resection with visible evidence of cancer at the resection margin.

### 2.2 Study Endpoints

The primary study endpoint aimed to elucidate the prognostic factors and overall survival and recurrence free survival of PACC through a comparison between the resectable PACC and PDAC groups after PSM. The secondary study endpoint was to investigate the clinical characteristics, metastatic sites, and treatment modalities by comparing the resectable and unresectable PACC groups.

### 2.3 Propensity Score Matching (PSM) Strategy

PSM was employed to mitigate selection bias when estimating the causal treatment effects and to achieve a balance in potential confounders between the study group of PACC and the control group of PDAC. An individual propensity score was generated through logistic regression modeling, based on seven covariates: tumor size, lymph node involvement, lymphovascular invasion (LVI), perineural invasion (PNI), tumor stage, radicality, and surgical procedures such as pancreaticoduodenectomy (PD) and distal pancreatectomy (DP). The rationale for using these covariates in this PSM study was these factors were commonly used in predicting the prognosis of PDAC. Subsequently, patients from the PACC study group and the PDAC control groups were paired in a 1:3 ratio, with matches starting from cases with the largest propensity score. A specific caliper width of 0.04 standard deviations of the logit of the estimated propensity score was applied.

### 2.4 Statistical Analysis

Statistical analyses were conducted using the Statistical Product and Service Solutions 26.0 version (IBM Corp., Armonk, NY, USA). Categorical variables are expressed as numbers (percentages) and compared using Pearson's *χ* ^2^ test or Fisher's exact test for contingency tables. Continuous data are presented as median (range) and mean ± standard deviation. Student's *t*‐test was employed to compare the means of two groups. In cases where continuous variables did not follow a normal distribution, the Wilcoxon rank‐sum test was utilized. Significance was determined by using a two‐sided test. A *p*‐value of < 0.05 was considered statistically significant.

## 3 Results

**Figure 1:**
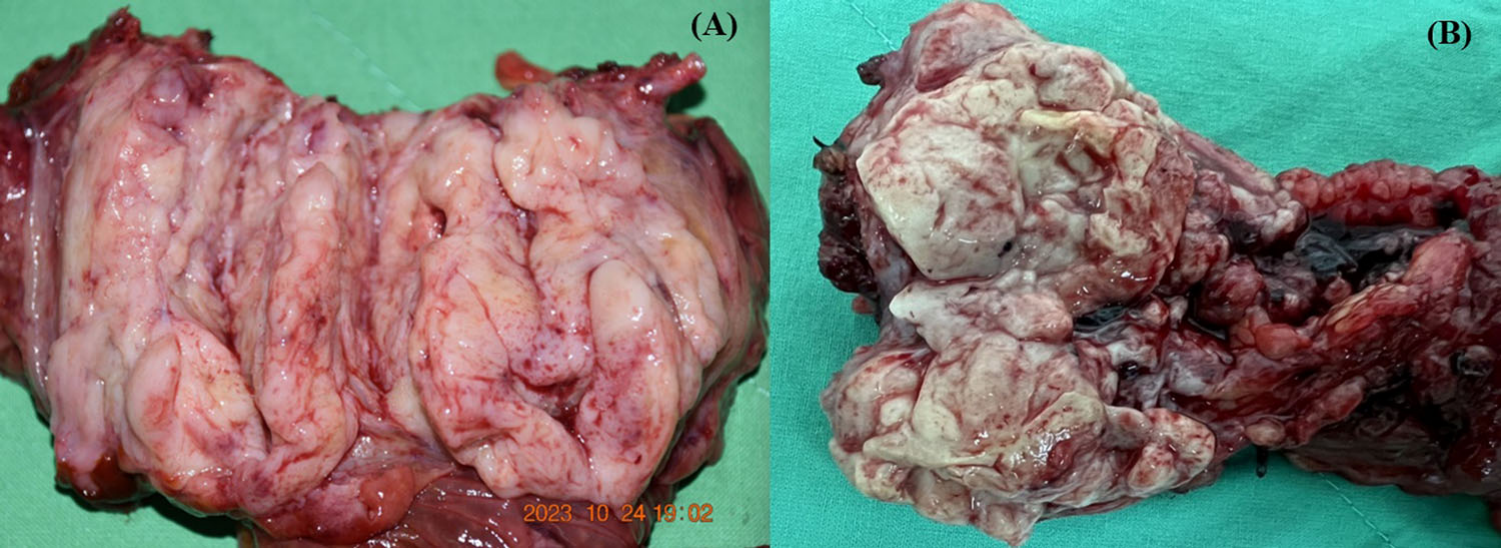
Gross pictures of pancreatic acinar cell carcinoma from (A) a patient undergoing pancreaticoduodenectomy, and (B) another patient undergoing distal pancreatectomy typically present a large, well‐circumscribed mass with fleshy consistency and at least partial encapsulation.

Twenty patients with PACC were enrolled in the study, comprising 11 resectable and nine unresectable cases (Table 1). Figure 1 illustrates gross pictures of pancreatic acinar cell masses, typically presenting as large, well‐circumscribed masses with a fleshy consistency and at least partial encapsulation. Female patients predominated (75%). The median age of PACC patients was 67 years, ranging from 19 to 86 years. The majority of PACCs were located in the pancreatic head (60%) compared to the body‐tail (40%). The median tumor size of PACC was 5.9 cm, ranging from 1.5 to 19.5 cm. The most common clinical presentation was epigastric pain (55.0%), followed by weight loss (45.0%) and jaundice (30.0%). Only one patient (5.0%) presented with Schmid's triad, including subcutaneous fat nodular necrosis, polyarthritis and eosinophilia. Serum lipase elevation without an association of elevated serum amylase was noted in 9 (64.3%) out of 14 PACC patients, with 66.7% in the resectable group and 60.0% in the unresectable group (*p* = 0.903). Serum CA 19‐9 was elevated in 47.4% (9/19) of PACC patients, and serum AFP was elevated in 44.4% (4/9) of PACC patients. There were no significant differences regarding sex distribution, age, tumor location, tumor size, clinical presentation, and elevation of serum markers including lipase, amylase, CEA, and AFP between resectable and unresectable groups, except for more patients presenting with body weight loss (18.2% vs. 77.8%, *p* = 0.008) and elevated serum CA 19‐9 (27.3% vs. 75.0%, *p* = 0.040) in the unresectable group.

**Table 1:** Demographics for patients with pancreatic acinar cell carcinoma.

	Total PACC	Resectable PACC	Unresectable PACC	*p*‐Value
Patients, *n* (%)	20	11 (55.0%)	9 (45.0%)	
Sex				0.436
Female	15 (75.0%)	9 (81.8%)	6 (66.7%)	
Male	5 (25.0%)	2 (18.2%)	3 (33.3%)	
Age, year old				0.087
Median (range)	67 (19–86)	63 (19–76)	68 (51–86)	
Mean ± SD	63.7 ± 14.7	58.6 ± 15.8	69.9 ± 10.9	
Location of PACC				
Head	12 (60.0%)	7 (63.6%)	5 (55.6%)	0.714
Body‐tail	8 (40.0%)	4 (36.4%)	4 (44.4%)	
Tumor size, cm				0.154
Median (range)	5.9 (1.5–19.5)	4.5 (1.5–13.0)	6.8 (2.9–19.5)	
Mean ± SD	6.7 ± 4.0	5.5 ± 3.0	8.1 ± 4.8	
Clinical presentations				
Epigastric pain	11 (55.0%)	6 (54.5%)	6 (55.6%)	0.964
Body weight loss	9 (45.0%)	2 (18.2%)	7 (77.8%)	0.008
Jaundice	6 (30.0%)	5 (45.5%)	1 (11.1%)	0.095
Nausea/vomiting	1 (5.0%)	1 (9.1%)	0	0.353
Diabetes mellitus	3 (5.0%)	2 (18.2%)	1 (11.1%)	0.660
Fever	1 (5.0%)	1 (9.1%)	0	0.353
Elevation of lipase only, *n* = 14	9 (64.3%)	6 (66.7%)	3 (60.0%)	0.903
Schmid's triad	1 (5.0%)	0	1 (11.1%)	0.450
Subcutaneous fat nodular necrosis	1 (5.0%)	0	1 (11.1%)	0.450
Polyarthritis	1 (5.0%)	0	1 (11.1%)	0.450
Eosinophilia	1 (5.0%)	0	1 (11.1%)	0.450
No symptom	2 (10.0%)	2 (18.2%)	0	0.479
Elevation of Lipase, *n* = 14	10 (71.4%)	7 (77.8%)	3 (60.0%)	0.480
Elevation of Amylase, *n* = 14	1 (7.1%)	1 (11.1%)	0	0.439
Elevation of CA 19‐9, *n* = 19	9 (47.4%)	3 (27.3%)	6 (75.0%)	0.040
Elevation of CEA, *n* = 19	5 (26.3%)	2 (18.2%)	3 (37.5%)	0.325
Elevation of AFP, *n* = 9	4 (44.4%)	3 (42.9%)	1 (50.0%)	0.858

Abbreviations: AFP, α‐fetoprotein; CA 19‐9, carbohydrate antigen 19‐9; CEA, carcinoembryonic antigen; PACC, pancreatic acinar cell carcinoma; SD, standard deviation.

The most common metastatic site of PACC was liver (35.0%), followed by peritoneal seeding, lung, spleen, bone, and adrenal gland (Table 2). Distant lymph node metastasis was observed in 20.0% of overall PACC patients and 44.4% in unresectable PACC patients. Regional lymph node involvement was noted in 65.0% of overall PACC patients, 36.4% in resectable, and 100% in unresectable PACC patients (*p* = 0.003). Among the 11 resectable PACC patients, 63.6% underwent pancreaticoduodenectomy and 36.4% underwent distal pancreatectomy. Overall, chemotherapy was administered in 78.9% of PACC patients, and radiotherapy in 5.3%.

**Table 2:** Metastasis and treatment for pancreatic acinar cell carcinoma.

	Total PACC	Resectable PACC	Unresectable PACC	*p*‐Value
Patients, *n* (%)	20	11 (55.0%)	9 (45.0%)	
Metastatic site, *n* = 20				
liver	7 (35.0%)	1 (9.1%)	6 (66.7%)	0.017
Peritoneal seeding	5 (25.0%)	0	5 (55.6%)	0.008
Lung	4 (20.0%)	0	4 (44.4%)	0.026
Spleen	3 (15.0%)	0	3 (33.3%)	0.074
Bone	2 (10.0%)	0	2 (22.2%)	0.189
Adrenal	1 (5.0%)	0	1 (11.1%)	0.450
Distant lymph node	4 (20.0%)	0	4 (44.4%)	0.026
Regional lymph node	13 (65.0%)	4 (36.4%)	9 (100%)	0.003
LVI, *n* = 12	5 (41.7%)	4 (36.4%)	1 (100%)	0.417
PNI, *n* = 12	6 (50.0%)	5 (45.5%)	1 (100%)	> 0.99
Treatment				
Resection, *n* = 11	11 (55.0%)	11 (100%)		NA
Pancreaticoduodenectomy	7 (35.0%)	7 (63.6%)		
Distal pancreatectomy	4 (20.0%)	43 (36.4%)		
No resection, *n* = 11	9 (45.0%)		9 (100%)	NA
Chemotherapy, *n* = 19	15 (78.9%)	9 (90.0%)	6 (66.7%)	0.213
Radiotherapy, *n* = 19	1 (5.3%)	0	1 (11.1%)	0.474
No chemo‐radiotherapy, *n* = 19	5 (26.3%)	1 (10.0%)	4 (44.4%)	0.089

Abbreviations: LVI, lymphovascular invasion; NA, not available; PACC, pancreatic acinar cell carcinoma; PNI, perineural invasion.

The median overall survival time was 20.0 months for the resectable PACC group and 6.7 months for the unresectable group, with a statistically significant difference (*p* < 0.001) (Table 3). The 1‐, 3‐, and 5‐year overall survival rates were 100%, 49.1%, and 32.0% respectively in resectable PACC patients, compared to 33.3%, 0%, and 0% in unresectable PACC patients. Four patients presented recurrence in the resectable PACC group, and the first site of recurrence were local recurrence in 2 and liver metastasis in 2. The 1‐, 3‐, and 5‐year recurrence free survival rates were 81.8%, 49.1%, and 49.1% respectively in resectable PACC patients.

**Table 3:** Survivals for pancreatic acinar cell carcinoma.

	Total PACC	Resectable PACC	Unresectable PACC	*p*‐Value
Patients, *n* (%)	20	11 (55.0%)	9 (45.0%)	
Overall survival time, month				< 0.001
Median	16.1	20.0	6.7	
Range	0.7–148.0	12.5–148.0	0.7–22.7	
Mean ± SD	27.0 ± 35.2	41.2 ± 42.5	9.6 ± 7.9	
1‐year overall survival	70.0%	100%	33.3%	
3‐year overall survival	23.3%	49.1%	0	
5‐year overall survival	15.6%	32.7%	0	
Recurrence free survival time, month				
Median	16.1	20.0	0	
Range	0.7–148.0	10.8–148.0	0	
Mean ± SD	27.0 ± 35.2	40.2 ± 43.0	0	
1‐year recurrence free survival	70.0%	81.8%	0	
3‐year recurrence free survival	23.3%	49.1%	0	
5‐year recurrence free survival	15.6%	49.1%	0	

Abbreviations: PACC, pancreatic acinar cell carcinoma; SD, standard deviation.

**Figure 2:**
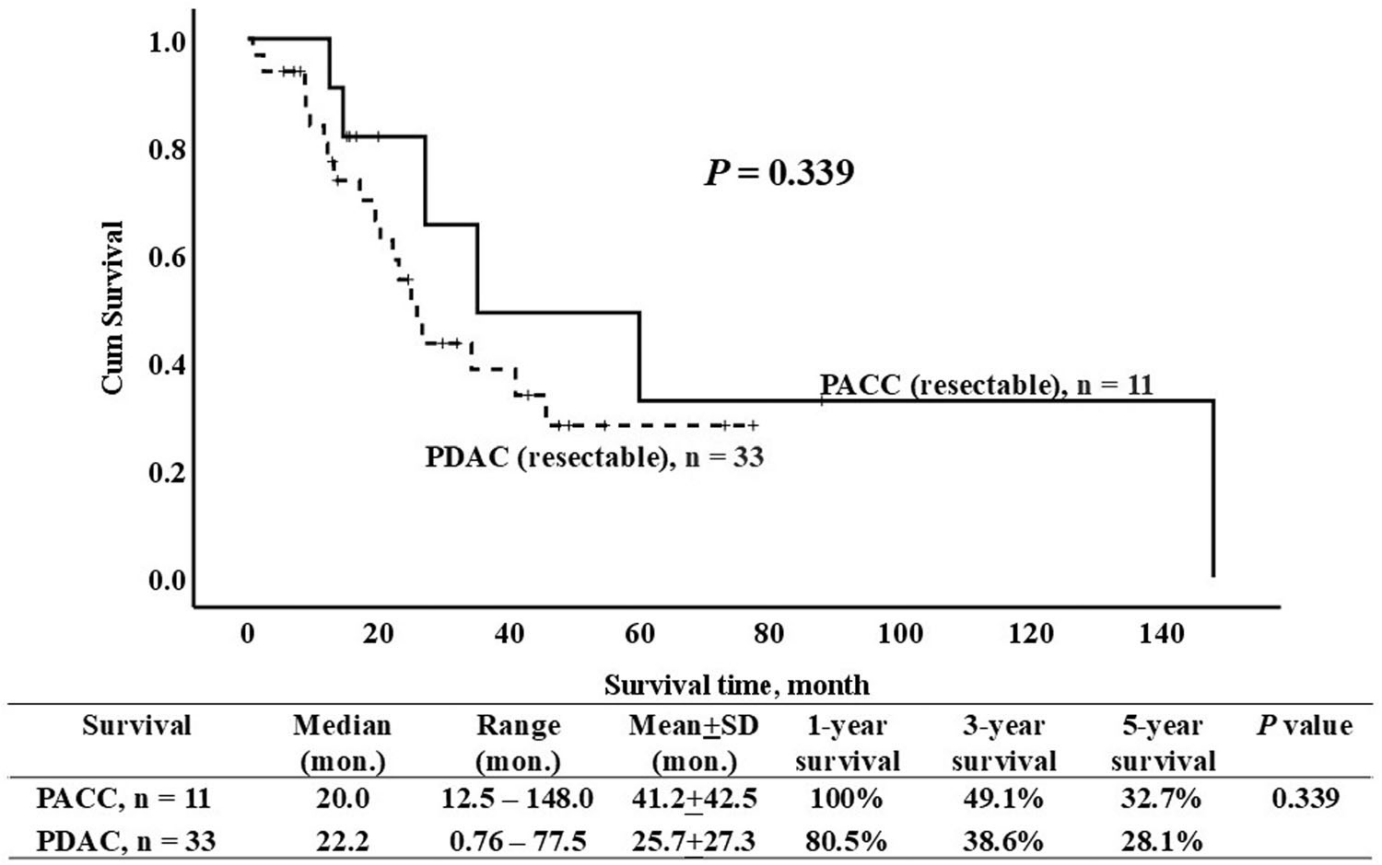
Survival curves for resectable pancreatic acinar carcinoma (PACC) and pancreatic duct adenocarcinoma (PDAC) after propensity score matching with a 1:3 ratio.

A total of 353 patients with resectable PDAC, including 215 undergoing pancreaticoduodenectomy and 138 distal pancreatectomy, were included as the control group to assess the prognosis of PACC. Comparing resectable PACC with the PDAC group, there was a lower rate of lymph node involvement (36.4% vs. 66.1%, *p* = 0.042), LVI (36.4% vs. 72.8%, *p* = 0.008), and PNI (45.5% vs. 86.0%, *p* < 0.001) in the resectable PACC group (Table 4). After PSM based on seven covariates commonly used to predict the prognosis of PDAC, including tumor size, lymph node involvement, LVI, PNI, tumor stage, radicality, and surgical procedure, there was no significant difference in survival between resectable PACC and PDAC (Figure 2).

**Table 4:** Comparison of prognostic factors between resectable PACC and PDAC.

	Resectable PACC	Resectable PDAC	*p*‐Value
Patients, *n* (%)	11	353	
PD cases	7	215	
DP cases	4	138	
Tumor size, cm			0.765
Median (range)	4.5 (1.5–13.0)	4.2 (4.0–8.0)	
Mean ± SD	5.5 ± 3.0	5.1 ± 1.9	
Lymph node involvement	4 (36.4%)	226 (66.1%)	0.042
LVI, *n* = 12	4 (36.4%)	249 (72.8%)	0.008
PNI, *n* = 12	5 (45.5%)	294 (86.0%)	< 0.001
Stage			0.343
I	1(9.1%)	93 (27.2%)	
II	8 (72.7%)	169 (49.4%)	
III	1 (9.1%)	63 (18.4%)	
IV	1 (9.1%)	17 (5.0%)	
Radicality			0.130
R0	11 (100%)	283 (82.7%)	
R1 + 2	0	59 (17.3%)	

Abbreviations: DP, distal pancreatectomy; LVI, lymphovascular invasion; PACC, pancreatic acinar cell carcinoma; PD, pancreaticoduodenectomy; PDAC, pancreatic duct adenocarcinoma; PNI, perineural invasion; SD, standard deviation.

## 4 Discussion

PACC, in comparison with PDAC, represents a relatively rare pancreatic malignancy characterized by distinct clinical and morphological features. One notable characteristic is the elevation of serum lipase alone without an association with elevated serum amylase levels. Serum lipase elevation could be observed in 24%–58% of patients with PACC [1]. Given its specificity, an elevated serum lipase level serves as a valuable aid in distinguishing PACC from other pancreatic malignancies. In our study, isolated elevation of serum lipase without an associated elevation of serum amylase was observed in 64.3% of our PACC patients, higher that those reported in the literature. Excessive expression of serum lipase, reaching levels as high as 10,000 U/dL, can lead to lipase hypersecretion syndrome, characterized by subcutaneous fat necrosis, polyarthralgia, and eosinophilia, often referred to as “Schmid's triad”. This triad is associated with a poor prognosis [2, 4, 5, 12]. Though Schmid's triad is typical for PACC, it is usually very rare, about 0%–15% [5, 13]. In our study, Schmid's triad was observed in only 1 (5.0%) of all PACC patients, with none in the resectable group and 11.1% in the unresectable group. In contrast to PDACs, PACCs typically present as large masses at diagnosis, with a median size of approximately 4.5–5.5 cm in resected cases. They often exhibit an ovoid shape and are well‐circumscribed, characterized by fibrous septa and a high amount of tumor cells, but with minimal stroma and no desmoplasia, [1, 3, 6, 8, 9, 14] as seen in two of our cases. One‐third of patients with PACC may exhibit calcifications. Additionally, the tumor often demonstrates heterogeneous enhancement following intravenous infusion of contrast material. Some studies have suggested that when encountering an ovoid, exophytic, and hypodense large mass with a well‐defined enhancing capsule arising from the pancreas, and without appreciable biliary or pancreatic dilation in imaging studies, PACC should be considered in the list of differential diagnoses [2, 5, 14, 15, 16]. The median size of PACC in this study was 5.9 cm among all patients, with sizes of 4.5 cm in the resectable group and 6.8 cm in the unresectable group, aligning with findings reported in the literature [1, 3, 6, 9].

PACC can manifest in any region of the pancreas, although it most frequently occurs in the head. While commonly diagnosed in individuals in their 60 s, it can also occur in pediatric patients [1, 2, 3, 7, 17]. Our findings align with literature reports, indicating a median age of 67 years, with ages ranging from 19 to 86 years, and with 60.0% of cases located in the pancreatic head. PACC is reported to occur more frequently in males [1, 2, 10]. However, our study reveals a predominance of females, with a distribution of 75% compared to 25% males. In a comparative study by Yonkus et al. [3] involving 589 PACC and 137,962 PDAC patients identified from the National Cancer Data Base and Surveillance Epidemiology and End Results datasets, several significant differences were observed between PACC and PDAC. Patients with PACC were more likely to be male (72.8% vs. 51.7%) and tended to be younger at the time of diagnosis (64.0 vs. 68.0 years old). Patients with PACC were more prone to nodal involvement (49.3% vs. 28.2%) and were less frequently located in the head of the pancreas (44.5% vs. 51.7%). Huang et al. [6] conducted a retrospective analysis comparing 52 PACC and 355 PDAC patients undergoing surgical interventions, revealing comparable clinical characteristics between the two groups, except for a lower mean age observed in PACC compared to PDAC (mean: 50.8 ± 10.9 vs. 59.4 ± 10.9 years).

Clinical symptoms of PACC closely resemble those of classical PDAC, with the exception of rare occurrences of pancreatic panniculitis associated with elevated serum lipase levels. A comprehensive review by Calimano‐Ramirez et al. [2] demonstrated that most PACC cases present with vague symptoms, including abdominal pain (60%), weight loss (45%), nausea and vomiting (20%), and diarrhea (8%). Unlike PDAC, PACC infrequently obstructs the bile duct, leading to jaundice in only 12% of cases. This distinction arises from the different growth pattern of PACC, which typically involve pushing rather than infiltrating adjacent structures [2, 17, 18]. In our study, we observed that the most common clinical presentation is epigastric pain (55.0%), contrasting with jaundice (30%). Another notable feature is the relatively high prevalence of elevated serum AFP, ranging widely from 7.5% to 47% according to some studies [1, 6, 13]. In our cohort, elevation of serum AFP was noted in as high as 44.4% of total PACC patients, with rates of 42.9% in resectable and 50.0% in unresectable PACCs.

Resectability emerges as the most crucial factor determining survival in individuals with PACC [1, 2, 7, 10]. Indeed, patients undergoing tumor resection have consistently exhibited significantly improved survival outcomes compared to those with unresectable disease or who are not surgical candidates [8, 10]. This is also evidenced in this study, wherein a median survival of 20.0 months was observed in resectable PACC patients, contrasting with 6.7 months in unresectable cases. Furthermore, the 1‐, 3‐, and 5‐year survival rates were notably higher in resectable PACC patients, at 100%, 49.1%, and 32.0%, respectively, compared to 33.3%, 0%, and 0% for the unresectable cases in this study. Huang et al. [6] also reported impressive survival rates, with 1‐, 3‐, and 5‐year survival rates of 96.7%, 71.7%, and 31.8%, respectively, and a median survival time of 48 months among resectable PACC patients. These findings strongly indicate that radical resection is associated with prolonged overall survival. Takahashi et al. [19] reported that the most common metastatic sites include the liver (68%), peritoneum (19%), and distant lymph nodes (14%). Our study corroborates these findings, with the most common metastatic site being the liver (35.0%), followed by the peritoneum (25.0%), distant lymph nodes (20.0%), and lung (20.0%).

PACC represents a tumor entity characterized by a less aggressive growth pattern and has been reported to carry a more favorable prognosis compared to PDAC. Resected PACC patients have been reported to exhibit 5‐year survival rates ranging from 36% to 72% and median survival times spanning from 18 to 123 months [1, 2, 3, 8, 10, 11, 20]. Nevertheless, Huang et al. [6] discovered that LVI (23.1% vs. 35.5%, *p* = 0.044) and PNI (7.7% vs. 56.1%, *p* < 0.001) were less prevalent in PACC patients compared to PDAC. Additionally, Schmidt et al. [20] found that PACC exhibited fewer instances of nodal metastasis, with rates of 32.1% compared to 48.0% in PDAC. However, since these reports primarily stem from large registry‐based analyses or limited patient series, it is inevitable that selection bias may influence the comparison of prognosis between PACC and PDAC. In this study, we also demonstrated that lymph node involvement (36.4% vs. 66.1%, *p* = 0.042), LVI (36.4% vs. 72.8%, *p* = 0.008), and PNI (45.5% vs. 86.0%, *p* < 0.001) were less prevalent in PACC patients compared to PDAC. Furthermore, after PSM based on seven covariates commonly used to predict the prognosis of PDAC, no significant survival difference was observed between PACC and PDAC in resected patients. These findings imply that the superior survival outcome observed in PACC patients, when compared with PDAC, might be attributed to a lesser association with poor prognostic factors such as lymph node involvement, LVI, and PNI.

There are some limitations to this study. This is a retrospective cohort study with a very limited number of patients spanning over a 20‐year period. Therefore, selection bias is inevitable, despite attempts to mitigate it through propensity score matching, which aims to mimic some characteristics of a randomized controlled trial.

## 5 Conclusion

In conclusion, PACC emerges as a relatively uncommon pancreatic malignancy characterized by distinct clinical features, notably including elevated serum lipase without associated amylase elevation. Typically, it presents as a large, well‐circumscribed mass with fleshy consistency and partial encapsulation. Resectability stands out as the most critical determinant of survival for PACC patients. PACC is associated with lower rates of lymph node involvement, LVI, and PNI, which might attribute to superior outcome when compared with PDAC reported in the literature. However, there is no survival difference between resecrable PACC and PDAC after PSM.

## Author Contributions

**Bor‐Shiuan Shyr:** conceptualization, methodology, data curation, writing–review and editing, writing–original draft, visualization, formal analysis, project administration, resources, software, validation. **Ting‐Chung Chen:** conceptualization, methodology, data curation, investigation, validation, formal analysis, writing–review and editing, writing – original draft, resources, project administration. **Shin‐E Wang:** conceptualization, methodology, software, data curation, investigation, validation, formal analysis, funding acquisition, supervision, project administration, visualization. **Shih‐Chin Chen:** conceptualization, methodology, data curation, investigation, validation, supervision. **Yi‐Ming Shyr:** conceptualization, methodology, software, data curation, investigation, validation, formal analysis, supervision, funding acquisition, resources. **Bor‐Uei Shyr:** conceptualization, methodology, software, data curation, investigation, validation, formal analysis, supervision, funding acquisition, visualization, project administration, resources, writing – original draft, writing – review and editing.

## Conflicts of Interest

The authors declare no conflicts of interest.

## Transparency Statement

The lead author Bor‐Uei Shyr affirms that this manuscript is an honest, accurate, and transparent account of the study being reported; that no important aspects of the study have been omitted; and that any discrepancies from the study as planned (and, if relevant, registered) have been explained.

## Data Availability

The data that support the findings of this study are available from the corresponding author upon reasonable request.
